# PS4DR: a multimodal workflow for identification and prioritization of drugs based on pathway signatures

**DOI:** 10.1186/s12859-020-03568-5

**Published:** 2020-06-05

**Authors:** Mohammad Asif Emon, Daniel Domingo-Fernández, Charles Tapley Hoyt, Martin Hofmann-Apitius

**Affiliations:** 1 Department of Bioinformatics, Fraunhofer Institute for Algorithms and Scientific Computing (Fraunhofer SCAI), 53757 Sankt Augustin, Germany; 2 Bonn-Aachen International Center for IT, Rheinische Friedrich-Wilhelms-Universität Bonn, 53117 Bonn, Germany

**Keywords:** Drug repositioning, Drug discovery, Multi-omics, Pathways, Software, Bioinformatics

## Abstract

**Background:**

During the last decade, there has been a surge towards computational drug repositioning owing to constantly increasing *-omics* data in the biomedical research field. While numerous existing methods focus on the integration of heterogeneous data to propose candidate drugs, it is still challenging to substantiate their results with mechanistic insights of these candidate drugs. Therefore, there is a need for more innovative and efficient methods which can enable better integration of data and knowledge for drug repositioning.

**Results:**

Here, we present a customizable workflow (*PS4DR)* which not only integrates high-throughput data such as genome-wide association study (GWAS) data and gene expression signatures from disease and drug perturbations but also takes pathway knowledge into consideration to predict drug candidates for repositioning. We have collected and integrated publicly available GWAS data and gene expression signatures for several diseases and hundreds of FDA-approved drugs or those under clinical trial in this study. Additionally, different pathway databases were used for mechanistic knowledge integration in the workflow. Using this systematic consolidation of data and knowledge, the workflow computes pathway signatures that assist in the prediction of new indications for approved and investigational drugs.

**Conclusion:**

We showcase *PS4DR* with applications demonstrating how this tool can be used for repositioning and identifying new drugs as well as proposing drugs that can simulate disease dysregulations. We were able to validate our workflow by demonstrating its capability to predict FDA-approved drugs for their known indications for several diseases. Further, *PS4DR* returned many potential drug candidates for repositioning that were backed up by epidemiological evidence extracted from scientific literature. Source code is freely available at https://github.com/ps4dr/ps4dr.

## Background

De novo drug discovery remains a time-consuming, costly, and failure-prone process, despite advances in high-throughput data generation techniques and analytical approaches. On average, it takes approximately 10 to 15 years and 1.5 billion dollars to bring a drug to market [1]. While traditional drug discovery research is able to propose numerous candidate drugs, the majority of them fail in clinical trials due to lack of efficacy or undesired effects in these trials [2]. Therefore, drug repositioning has emerged as an alternative in drug discovery research [3] that hinges on identifying new indications for investigational or approved drugs in order to reduce the time and cost of pre-clinical development and primary stages of clinical trials.

Computational drug repositioning methods have recently become popular due to the increased availability of drug-related *-omics* data through sources like CMap (Connectivity Map [4]) and LINCS (Library of Integrated Network-Based Cellular Signatures [5]) (see Tanoli et al. [6] for a review on databases and methods). In recent years, they have evolved to accommodate and utilize novel high-throughput data such as genetic [7], chemical [8], pharmacological [9], and clinical [10]. Computational drug repositioning methods can be categorized as (i) drug-based, where knowledge comes from the chemical or pharmaceutical perspective, or (ii) disease-based, where the strategy focuses on different aspects of the disease, such as symptomatology or pathology [11]. Following, we outline methods from both categories that involve the usage of transcriptomics and GWAS data for drug repositioning purposes.

Transcriptomics data has historically been used to unravel the molecular mechanisms of complex diseases [12–14]. Accordingly, numerous drug repositioning approaches have relied on contrast experiments of transcriptomics readouts such as disease samples, drug perturbed cells and animal models to identify drugs that revert the signature of the disease and eventually its pathogenic phenotype to ultimately predict new indications for existing drugs [4, 15, 16]. To facilitate novel approaches that could systematically exploit this concept, Lamb et al. [4] developed a comprehensive catalog of small molecule perturbed gene expression signatures called CMap. They demonstrated that gene expression signatures can be used to identify drugs with shared mechanisms of action (MoAs), discover unknown MoAs of drugs, and propose potential new therapeutics. Furthermore, a variant of the CMap method was later used by Sirota et al. [16] to compare disease gene signatures against drug-induced gene expression signatures to score each drug-disease pair based on their similarity profile for drug repositioning.

However, the high dimensionality of gene expression signatures has motivated the use of network-based analysis to assist in the interpretation of biological processes which are perturbed by a given drug. Not only are these analyses instrumental in determining relevant molecular signatures as markers of phenotypes but also in garnering novel mechanistic insights into various biological functions and disease. For example, Iorio et al. [15] used Gene Set Enrichment Analysis (GSEA [17]) to build a drug similarity network from the distances of the GSEA scores for each drug pair in order to investigate the biological processes enriched in a set of drug subnetworks to identify compounds with similar MoAs. Suthram et al. [18] integrated disease gene expression signatures with large scale protein-protein interaction networks to identify disease similarities. They discovered a set of common pathways and processes which were dysregulated in most of the investigated diseases and that could be targeted by the drugs indicated for other diseases. Keiser et al. [19] showed that drug-target interaction networks could be used to predict off-targets for known drugs by comparing the similarity of the ligands that bind to the corresponding targets.

Single nucleotide polymorphisms (SNPs) have gained attention in biomedical research due to the impact of genetic variations in numerous complex diseases. Although the majority of SNPs do not have an effect on the phenotypic outcome, some might be directly involved in disease etiology by affecting the associated gene’s function depending on their occurrence in the genomic loci. Therefore, identifying disease-associated SNPs via genetic studies (e.g., GWAS) and targeting the corresponding genes has become a common practice for generating hypotheses to investigate molecular mechanisms of disease. Accordingly, new methods are being developed to incorporate GWAS knowledge in the drug repositioning domain. For instance, Sanseau et al. [7] collected disease-associated genes from the GWAS Catalog [20] and evaluated whether these genes were targeted by drugs. In their post hoc analysis, they observed that these genes were more likely to be a drug target than housekeeping genes. They mapped GWAS genes to the genes which were targeted by drugs listed in the pharmaprojects database (http://www.pharmaprojects.com/) and later proposed that drugs with indications different from the GWAS traits could be of potential drug repositioning interest. In another instance, Lencz and Malhotra [21] used the results from large scale GWAS conducted by the Psychiatric Genomics Consortium–Schizophrenia Workgroup (PGC–SCZ) [22] to predict drug repositioning candidates in schizophrenia. First, they identified the overlap between the known drug targets from Rask-Andersen et al. [23] and potential schizophrenia candidate genes from GWAS. Next, they characterized the MoA of drugs targeting the overlapped genes to propose drugs for schizophrenia treatment. Further, Zhang et al. [24] illustrated another strategy to use GWAS data for prioritizing candidate genes from the GWAS identified loci for drug repositioning. They prioritized genes by scoring them with seven criteria such as cis-eQTL, text mining, and functional enrichment to propose new targets for colorectal cancer drug treatments.

While studies have leveraged transcriptomics and genetics data for prioritizing drug repositioning candidates independently, recent approaches have started to utilize them in combination with other data types. So et al. [25] proposed a framework for drug repositioning by combining GWAS-imputed transcriptome signatures and drug-induced changes in gene expression (CMap) in the field of psychiatric disorders. They imputed gene expression signatures from GWAS summary statistics instead of using expression data from microarray or RNA-sequencing studies and compared them with drug-induced expression changes. Zhang et al. [26] demonstrated another drug repositioning workflow by mining -*omics* data such as GWAS, proteomics, and metabolomics from publicly available sources to find diabetic risk proteins and then filtered them to druggable targets. They further analyzed the pathogenicity of these prioritized targets and found several drugs for these targets that have the potential for diabetic treatments. Later, Ferrero and Agarwal [27] presented a systematic approach which integrated GWAS data and gene expression signatures from diseases and drugs perturbation to generate drug repositioning hypotheses. They demonstrated that (i) GWAS-associated genes in disease are more likely to be differentially expressed in the same disease, and (ii) drug perturbed genes in disease are enriched for GWAS-associated genes in the same disease. They eventually proposed statistically significant drug-disease pairs from the latter analysis could be used for drug repositioning.

Above we surveyed the state-of-the-art in silico strategies for drug repositioning by using transcriptomics and GWAS data. However, there is a lack of systematic approaches that can integrate mechanistic knowledge from pathways with data from multiple modalities to ultimately provide a better understanding of the drug’s mechanism of action in the disease context. Therefore, we introduce *PS4DR,* a multimodal and integrative workflow that uses multiple data modalities (i.e., GWAS and transcriptomics) together with pathway knowledge to predict approved drugs in new indications. Finally, we show that our workflow is able to identify FDA-approved drugs for their known indications and predict new indications for existing drugs using publicly available datasets.

## Results

**Fig. 1:**
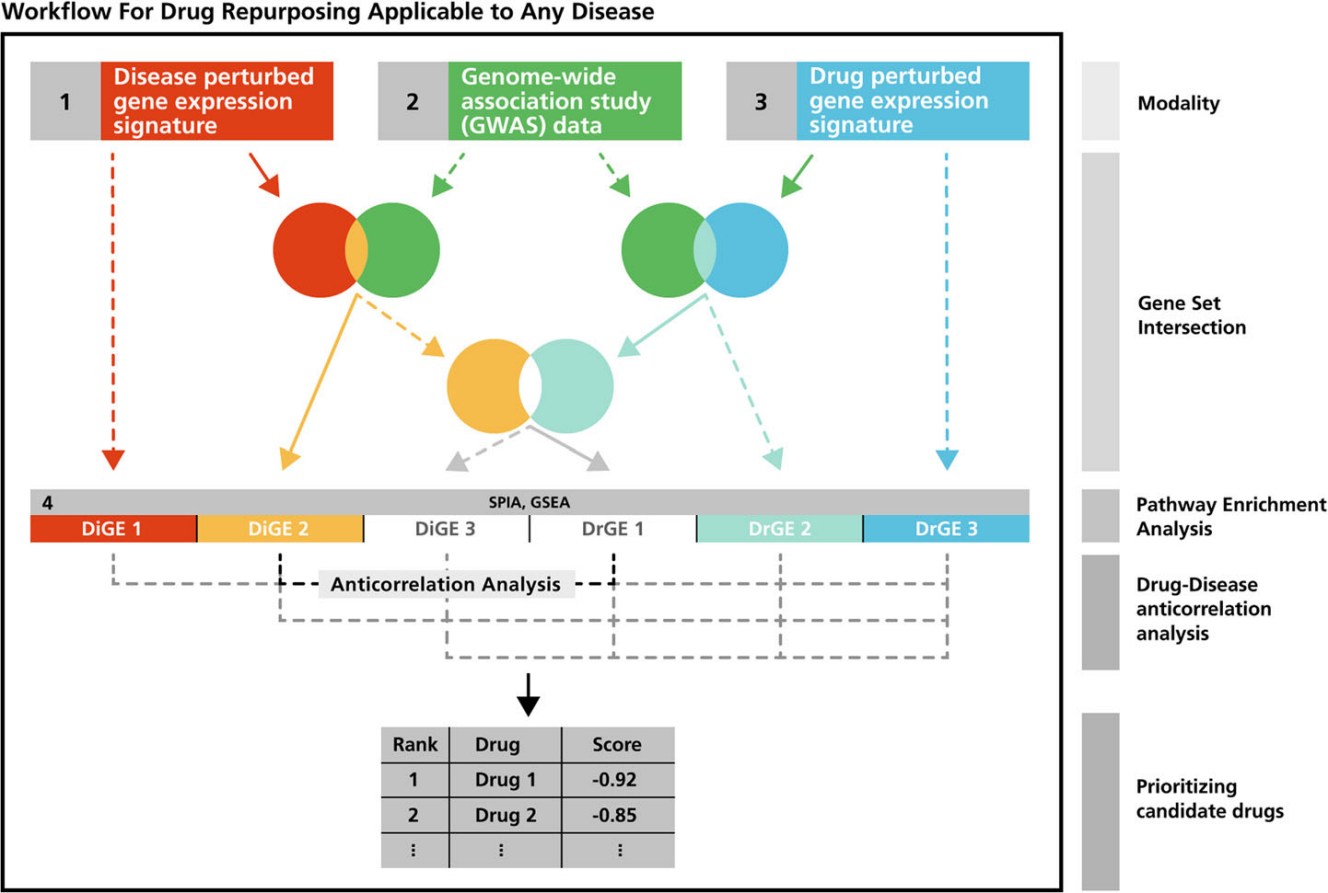
*An overview of the PS4DR workflow.* The workflow requires three different datasets as inputs, (i) disease perturbed gene expression signatures, (ii) genome-wide association study (GWAS) data, and (iii) drug perturbed gene expression signatures. The first and optional part of the workflow involves different filtering steps based on gene set intersection operations that enable the identification of genes in the gene expression signatures that have also been identified in a GWAS of the studied disease. To retain the maximum flexibility in the workflow, users can decide which of the filtering steps they wish to apply, if any. The next step uses the transcriptomics datasets, filtered or not, to conduct pathway enrichment analysis and evaluate the direction of perturbation for each affected pathway in a particular disease context. While the dotted lines in the figure represent all possible combinations of the filtering steps that can be applied and lead to the pathway enrichment step, solid lines show the option we chose to demonstrate the workflow. Finally, the last step uses the correlation of the pathway scores calculated by the previous step to prioritize drugs that are predicted to invert the pathway signatures observed in a given disease context

We developed *PS4DR,* an automated workflow that enables the integration of multimodal datasets together with pathway information from different canonical pathway databases to predict drug repositioning candidates in different diseases (Fig. 1). We showcase *PS4DR* using real-world gene expression signatures (i.e., Open Targets [28] and LINCS) and GWAS data (i.e., GWASdb [29], GWAS Catalog [20], GRASP [30], and PheWAS [31]). First, the workflow filters disease and drug transcriptomics (i.e., gene expression signatures) with the help of GWAS data. The next step involves calculating pathway signatures for diseases and drugs via pathway enrichment analysis with the filtered dataset. Finally, *PS4DR* performs an anti-correlation analysis by calculating correlation scores between the pathway signatures of drugs and diseases to prioritize drugs for each disease. Below, we show the utility of the workflow with three applications on how this tool can serve to i) identify drug repositioning candidates, ii) prioritize drug combinations, and iii) propose drugs that simulate disease dysregulations.

### Identifying drug repositioning candidates

As a first application, we explored the list of 26 diseases for which our workflow predicted drug repositioning candidates. While our workflow predicted plenty of drug candidates, we considered two criteria to prioritize predicted drugs. First, we prioritized all drugs in each disease based on their negative correlation scores. However, a drug could have a negative correlation score by only reverting a minority of the pathways dysregulated in the disease. Therefore, we also consider the relative number of the dysregulated pathways reverted by a drug for the prioritization process. While this prioritization approach facilitated narrowing down the candidate lists, we are aware that each of the drugs exhibiting negative correlation scores might have the potential to revert the disease condition even if they alter very few dysregulated pathways.

The distribution and Q-Q plots for the majority of the diseases that output drug predictions demonstrate that the correlation scores follow a normal distribution (Additional file: Fig. S1 and Fig. S2). Hence, we applied an arbitrary threshold to the correlation score to prioritize the proposed candidate drugs in each disease. We would like to point out that we used the same threshold for all diseases since we are exploring multiple indications; however, this threshold could be selected individually for each disease based on their underlying correlation score distributions. The applied threshold discarded drugs with a correlation score greater than − 0.4 or drugs which did not cover more than 50% of the affected pathways in the disease. This filtering step, intended to reduce the number of hits and facilitate the manual investigation of the results, returned a list of predicted drug candidates for 19 diseases (Additional file 1: Table S1). We further investigated the proposed drugs for five conditions to see whether *PS4DR* was able to identify FDA-approved drugs for their known indications and predict new indications for existing drugs in the prioritized list.

First, we focused on the predicted drug list for melanoma. We searched DrugBank [32] and scientific literature to collect evidence for the proposed drugs and summarized our findings in Table 1. Seven of nine predicted drugs are either already being used as cancer drugs or currently being studied in different clinical trials. This motivates further investigation of these drugs as repositioning candidates for the treatment of melanoma.

**Table 1:** Drug repositioning candidates for Melanoma. Drugs showing a negative correlation score less than or equal to − 0.40 and affecting more than 50% of the dysregulated pathways in melanoma. The last column outlines the current uses of the given drug in other conditions according to DrugBank and scientific literature

Drug	DrugBank ID	Correlation Score	Affected Pathways (%)	Description
**Crizotinib**	DB08865	−0.64	74.07	Used for the treatment of locally advanced or metastatic non-small cell lung cancer (NSCLC).
**Olmesartan**	DB00275	−0.85	55.56	Used for the treatment of hypertension.
**Sepantronium**	–	−0.21	74.07	Clinical trials in advanced non-small-cell lung cancer.
**Bortezomib**	DB00188	−0.52	62.96	Used for the treatment of multiple myeloma.
**Fluspirilene**	DB04842	−0.5	55.56	Used for the treatment of schizophrenia.
**Vistusertib**	DB11925	−0.44	66.67	Under investigation for the treatment of Advanced Gastric Adenocarcinoma.
**Olaparib**	DB09074	−0.44	66.67	A poly (ADP-ribose) polymerase (PARP) inhibitor indicated for the treatment of Ovarian and Breast Cancer.
**Tivozanib**	DB11800	−0.44	66.67	Used in trials for the treatment of solid tumors, Ovarian Cancer, Glioblastoma, Prostate Cancer among others.
**Belinostat**	DB05015	−0.43	55.56	Used for the treatment of patients with relapsed or refractory peripheral T-cell lymphoma (PTCL).

The topmost drug in our predicted shortlist, Crizotinib, a non-small cell lung cancer (NSCLC) drug, has been reported for its positive effect on melanoma by two studies [33, 34]. While Surriga et al. [33] suggested that Crizotinib could be used in adjuvant therapy for uveal melanoma due to its c-Met activity inhibition, recent research reported strong kinase fusion association with different melanoma subtypes [35] and encouraged the testing of kinase fusion inhibitor Crizotinib for melanoma treatment [34]. The third drug, Sepantronium, a selective small-molecule survivin suppressant, was reported to reduce the accumulation of survivin in G2/M mitotic arrest and induce apoptosis in human malignant melanoma cells in combination therapy with docetaxel [36, 37]. The following drug in Table 1, Bortezomib, is an approved drug for multiple myeloma that was suggested as a treatment for melanoma in combination therapy with temozolomide due to its ability to induce apoptosis and autophagic formation in human melanoma tumors [38, 39]. Another FDA approved drug Olaparib (for breast and pancreatic carcinoma), was also found to be effective against melanoma by inhibiting repair of single-strand DNA breaks in different combination therapies [40, 41].

The last two approved drugs in the list (i.e., Tivozanib for renal cell carcinoma and Belinostat for peripheral T-cell lymphoma) have been positively associated with a better response in melanoma [42, 43]. Moreover, another mTOR inhibitor drug, Vistusertib (AZD-2014), currently in phase II clinical trial for meningioma, was reported to have a positive impact by mTORC1/2 inhibition of the resistance to MAPK pathway inhibitors in melanomas with high oxidative phosphorylation [44, 45]. Interestingly, we also have two drugs, Olmesartan, for hypertension, and Fluspirilene, for schizophrenia, from very different therapeutic areas in our shortlist. While no reports of their potential role in melanoma treatment have been found yet, numerous studies have suggested their applicability in different cancer treatments [46–49].

We have found three drugs in breast carcinoma (Additional file 1: Table S1). The first drug, AT-7519, a selective inhibitor of specific Cyclin-Dependent Kinases (CDKs), is under investigation for the treatment of leukemia, lymphoma, myelodysplastic syndrome, and solid tumors [32]. This is in concordance with the study by Yu et al. [50] describing how a subgroup of breast cancer patients benefited from the treatment of CDK4 kinase inhibitors. The next drug, Omacetaxine Mepesuccinate, used for chronic myeloid leukemia, is in a clinical trial (NCT01844869) for treating advanced solid tumors (i.e., breast, lung, colorectal and melanoma). Finally, Rigosertib has shown potent antitumor activity in various preclinical models such as breast cancer and pancreatic cancer xenografts and is currently under clinical trial [51].

Similarly, we found that six out of eight drugs proposed for pancreatic carcinoma are either already being used in different cancers or have been suggested in the literature, as we discuss below (Additional file 1: Table S1). The first drug, Fenofibrate, an antilipemic agent, was reported to inhibit pancreatic cancer cell proliferation via activation of p53 mediated by upregulation of MEG3 [52]. The next drug, Menadione, was found to induce reactive oxygen species to promote apoptosis via redox cycling in pancreatic cells [53, 54]. Fluoxetine, originally an antidepressant agent, was also reported to work as a chemosensitizer and acts with other cancer drugs to overcome multidrug resistance in cancer cells [55]. An investigational cancer drug, Tosedostat, was found to be well-tolerated and clinically active against pancreatic ductal adenocarcinoma patients in phase I/II clinical trial ([56]; NCT02352831). Another drug, AZD-6482, a selective PI3Kβ inhibitor, could be useful in pancreatic cancer treatment because of its apoptotic effect in cancer cell lines [57]. Praziquantel was reported to inhibit cancer cell growth when used synergistically with paclitaxel via downregulating the expression of X-linked inhibitor of apoptosis protein (XIAP) [58].

While our workflow showed very promising results in cancer, we wanted to explore the results in complex disorders with no available treatments, such as Alzheimer’s disease (AD) and multiple sclerosis (MS). In the case of AD, the workflow provided fourteen shortlisted candidates (Table 2). The top drug on the list is Sirolimus (rapamycin), an immunosuppressant, already proposed for the treatment of AD by different studies [59–61]. It has been suggested that the therapeutic effect of this drug is due to the reduction of amyloid-beta levels caused by its inhibition of the mTOR signaling pathway [61]. Another compound, Pimozide, an antipsychotic agent, was recently suggested as a potential AD therapeutic which was reported to reduce toxic forms of tau protein by enhanced autophagy activity via AMPK-ULK1 axis stimulation [62]. Interestingly, we have two cancer drugs, Pevonedistat and Nilotinib, which could have potentially positive effects on AD treatment ([63–65]; NCT02947893). Pevonedistat, a neddylation inhibitor, could prevent neuronal damage and ameliorates cognitive deficits by preventing NRF2 protein degradation via inhibiting neddylation [63, 65]. Nilotinib, a tyrosine kinase inhibitor, has also been found to be very promising to delay the progression of AD by enhanced amyloid-beta clearance ([64]; NCT02947893).

**Table 2:** Drug repositioning candidates for Alzheimer’s disease (AD). Drugs showing a negative correlation score less than or equal to −0.40 and affecting more than 50% of the dysregulated pathways in AD

Drug	DrugBank ID	Correlation Score	Affected Pathways (%)
**Sirolimus (Rapamycin)**	DB00877	−0.69	66.67
**Pevonedistat**	DB11759	−0.66	60.61
**Nilotinib**	DB04868	−0.64	60.61
**Terfenadine**	DB00342	−0.57	57.58
**Doxylamine Succinate**	DB00366	−0.57	54.55
**Halcinonide**	DB06786	−0.57	51.52
**Promazine Hydrochloride**	DB00420	−0.53	66.67
**Mosapride**	DB11675	−0.45	60.61
**Pimozide**	DB01100	−0.45	57.58
**Ritanserin**	DB12693	−0.45	57.58
**Betamethasone**	DB00443	−0.44	66.67
**Cinacalcet Hydrochloride**	DB01012	−0.43	72.73
**Methapyrilene Hydrochloride**	DB04819	−0.43	72.73
**Trametinib**	DB08911	−0.40	60.61

Animal studies have demonstrated that the blockade of muscarinic receptors results in increased levels of acetylcholine and improve cognition [66]. Therefore, another proposed drug, Terfenadine which is a muscarinic receptor antagonist and has not yet been linked to AD, could be a potential repositioning candidate. Similarly, several 5-HT6R antagonists have advanced to different phases of clinical trials ([67]; NCT02258152; NCT02580305) as treatments for AD. The results also suggest another drug in the list, Ritanserin, that has not been directly indicated for AD. The high score proposed by our workflow to this serotonin receptor antagonist may be explained by its regulation of the neuronal cholinergic and glutamatergic pathways, both dysregulated in AD. Furthermore, there is increasing evidence showing that neuroinflammation significantly contributes to AD pathogenesis [68, 69]. Hence, it is not surprising to find two anti-inflammatory agents in our list (i.e., Betamethasone and Halcinonide) that could be worth investigating as potential repositioning drugs. Finally, Doxylamine Succinate, a neurotransmitter agent and histamine antagonist, is also a promising candidate since the beneficial effects of histamine antagonists in AD have been reported in multiple studies [70–72].

Finally, we investigated the top ranked drugs proposed by *PS4DR* for multiple sclerosis (MS). Ranked at the top of the list, *PS4DR* successfully recovered methylprednisolone, a corticosteroid with anti-inflammatory action prescribed to treat acute exacerbations in patients with MS [73] (Additional File 1: Table S1).

### Prioritizing drug combinations

Although we have illustrated that our workflow is able to identify candidate compounds for drug repositioning, combining multiple drugs can provide more benefits since the number of affected pathways can be increased by taking advantage of their synergistic effects. Therefore, we applied our workflow to all drug pair combinations in all diseases in order to identify therapies that could have a greater effect than single-drug treatments. For this application, we exclusively considered combinations of two drugs for two reasons: i) application of multiple drugs is usually counterproductive since it increases the number of side effects and ii) calculation time increases exponentially with an increasing number of drugs.

We investigated the predictions of our workflow in breast cancer to verify if we have more drugs with a good negative correlation score and affected pathways (%). While we had three drugs from our single-drug prediction approach, we were able to retrieve 489 drug pairs from the drug combination approach with the same thresholds. To facilitate manual investigation, we increased our threshold of correlation score to less than or equal to − 0.50 and affected pathways greater than or equal to 80% and were still able to retrieve 34 drug pairs (Additional file 1: Table S2). Here, all 19 new drugs in these 34 pairs are partnered with one of the top two drugs, AT-7519 or Omacetaxine Mepesuccinate, from the single-drug approach. Fourteen of the new drugs have partnered with both AT-7519 or Omacetaxine Mepesuccinate. While we have found literature evidence for the beneficial role of seven of these new drugs in the treatment of breast cancer, another six drugs are reported to have positive effects in other solid tumor based cancer treatment as described below. The third drug from the single-drug approach, Rigosertib, which was reported to have antitumor activity in breast cancer cell lines [51], has partnered with both AT-7519 or Omacetaxine Mepesuccinate. BGJ-398, a fibroblast growth factor receptor inhibitor in the list, significantly prevented the outgrowth of tumor organoids in metastatic breast cancers [74]. An approved cancer drug, Erlotinib Hydrochloride, epidermal growth factor receptor inhibitor, has shown a very positive response rate when treated combinedly with Capecitabine and Docetaxel in advanced breast cancer patients [75]. Another drug Selumetinib, a tyrosine kinase inhibitor, is currently being tested in several clinical trials (i.e., NCT03162627; NCT03742102; NCT02503358) for different cancer types, including breast cancer. TAK-715 is a p38 MAP kinase inhibitor in the list that cross-reacts with casein kinase ɛ (CKIɛ). Since CKIɛ mutations have been linked with the proliferation of different breast cancer cell lines, this drug could be explored to repurpose it for breast cancer treatment [76]. Another investigated drug, Tivantinib, has also shown positive effect on breast cancer model by reducing the metastasis via c-MET inhibition [77]. Megestrol Acetate, a progesterone receptor agonist, is under various clinical trials either alone or in combination with other cancer drugs for breast cancer treatment (i.e., NCT03306472 and NCT03024580).

AZD-1775, a drug that inhibits the G2–M cell-cycle checkpoint gatekeeper WEE1 kinase, has been used in multiple trials studying the treatment of lymphoma, ovarian cancer, and adult glioblastoma [32, 78]. Another drug, Axitinib, a selective vascular endothelial growth factor receptor (VEGFR) inhibitor, is under investigation in different clinical trials for various cancer types (i.e., NCT02129647; NCT03494816; NCT03472560). Moreover, four other drugs i,e., BMS-777607, PF-04217903, R-406, and Isotretinoin are reported to have positive effects in different solid tumor cancer types in different studies [32, 79–81].

### Proposing drugs that simulate disease pathway signatures

**Fig. 2:**
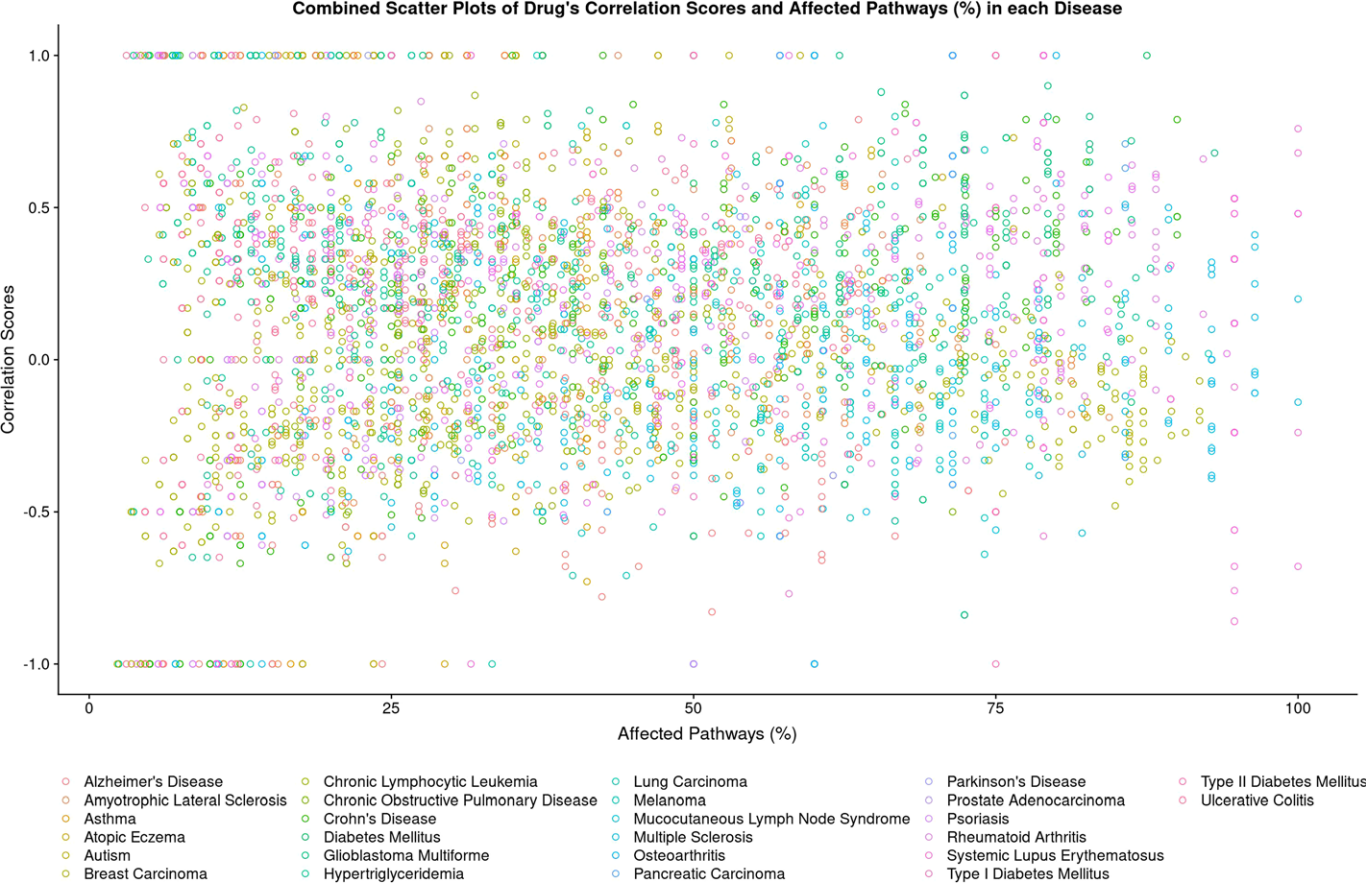
Combined scatter plots of the drug’s correlation scores against affected pathways (%) in each disease. The relative number of target pathways affected by the drug in the disease context is plotted along the x-axis and correlation scores on the y-axis. Drugs in the top-right corner of the plot might be interesting for developing in vitro disease models since this group of drugs shows positive correlation scores, covering a broad range of the affected pathways. The circles represent drugs and the color coding indicates their respective disease indication, as shown at the bottom

While we have initially focused on the drugs with the most negative correlation scores, we also anticipated a potential utility for drugs showing positive correlations. Well-characterized drugs with high positive correlation scores can provide information about how pathways or targets could be implicated in the molecular basis of the disease. Hence, as an extended application, the workflow may be used additionally as a prioritization tool to identify drugs that could be potentially employed to generate in-vitro or in-vivo models. By investigating the correlation scores (Fig. 2), researchers can readily identify drugs that could be used for this purpose. Our workflow predicted induction of disease pathway signatures for Pevonedistat in diabetes mellitus, Alvocidib in Crohn’s disease, and Entinostat and panobinostat in systemic lupus erythematosus (SLE) through very high positive correlation scores in addition to their broad coverage of affecting disease pathways. We see the need for further investigations of all the drugs with both high positive correlation scores and a high percentage of affected pathways for their use in potential disease model development.

## Discussion

Numerous innovative and interesting methods are constantly being developed to exploit high-throughput biological data in drug discovery research. However, there is still an urgent need for reproducible approaches which could systematically combine mechanistic knowledge with high-throughput data for drug repositioning purposes. In this work, we propose *PS4DR*, a drug repositioning workflow that combines data- and knowledge-driven information for predicting novel indications for prescribed drugs. We demonstrate the workflow using publicly available databases for disease and drug *-omics* data and employing pathway knowledge from various canonical pathway databases. The results show how *PS4DR* provides a comprehensive overview of the targeted pathways by drug or drug combinations and how this information can be useful to identify drug repositioning candidates. Finally, we validated the results of the workflow with epidemiological evidence extracted from the scientific literature to demonstrate that the workflow also prioritizes already approved drugs for numerous conditions.

However, our work is not without limitations, which we plan to address in future research. The connection between drug perturbed gene expression signatures, GWAS data, and disease-specific gene expression signatures is based on statistics derived from gene overlap. While the two latter datasets are disease-specific, drug-derived information is not contextualized. The linkage across the datasets could be more informative if there would be datasets available with drug perturbed gene expression signatures from disease models. Moreover, using advanced techniques such as deep learning [82] or network-based [83] methods to bridge different data modalities by inferring the association between heterogeneous features (i.e., genes, diseases) could also be viable alternative approaches to contextualize the data. Additionally, our workflow is limited to the availability of summarized disease- and drug- perturbed gene expression signatures. Finally, we would like to mention that the drug combination strategy approach is agnostic to other important processes such as kinetics, whether target genes are expressed in the tissue and whether the proposed drugs can be delivered to the tissue.

Although we applied the workflow to 43 diseases and 547 FDA approved and 126 investigational drugs (clinical trial phase I-III), the flexible design of the workflow allows for it to be run using any disease or drug for which GWAS and transcriptomics data is available. Similarly, other pathway databases could be used in the pathway enrichment step instead of the ones we are proposing. Therefore, we plan to use other datasets in the future such as DSigDB for drug-induced gene expression [84] as well as other pathway databases such as WikiPathways [85]. We also anticipate that incorporating new data modalities such as proteomics and eQTLs could be another prospect for enhancement of the workflow. While we have not considered drug side effects in our current work, integrating side effect information in a future extension could lead to better predictions. Moreover, we purposely restricted our analysis to exclusively approved drugs and those under clinical trial since our study was focused on finding repositioning drug candidates. However, the presented workflow could be applied to all LINCS drug perturbed gene expression signatures for drug discovery purposes. Running the workflow with novel datasets not only will provide new insights on candidate drugs but also allow to evaluate the reproducibility of the findings presented in this work.

## Conclusions

Here, we have presented *PS4DR,* a reproducible drug repositioning workflow that exploits multimodal datasets to predict drug candidates with the help of pathway knowledge. We have demonstrated how integrating pathway knowledge with transcriptomics and GWAS data can elucidate a drug’s mode of action in a disease condition as well as identify potential new applications for a drug. Our workflow predicted numerous drug candidates for several diseases which were validated with epidemiological evidence extracted from the literature and clinical trials. In addition, the modular design of the workflow enables investigators to choose any dataset from proprietary or public databases which suit their experimental needs. While the increased amount and dimensionality of personalized health data are improving health care, we hope our systematic approach to integrate contextual knowledge with data will pave the way towards mechanism-based drug repositioning in precision medicine research.

## Methods

Previous work from Ferrero and Agarwal [27] demonstrated that genes associated with a disease have a tendency to be differentially expressed both in a disease and drug context. Following their hypothesis, we propose a new workflow, *PS4DR*, that can exploit transcriptomics and GWAS data together with pathway knowledge to predict the drugs that best revert the pathway dysregulations observed in a given pathophysiological context. We compared the results generated using the PS4DR workflow with the drug-disease associations presented by Ferrero and Agarwal [27]. These results can be found in Additional file 1: Text Section 3.

In the following subsections, we describe our modular and flexible workflow (Fig. 1). We begin by introducing the different data modalities (e.g., GWAS, gene expression signatures, etc.) and the resources used in the workflow in the application scenario, followed by the data preprocessing steps. Finally, we discuss in detail the different components of the workflow, its implementation, and how it can be adapted to other software tools.

### Data modalities

*PS4DR* uses two different data modalities: GWAS and transcriptomics data. This section describes the datasets used for each modality for the case scenario. While we used various publicly available datasets as described below, users can use any other public or proprietary datasets of their preference in the workflow.

#### GWAS data

We have collected genetic association data from different publicly available GWAS datasets (i.e., GWASdb, GWAS catalog, GRASP, and PheWAS). We integrated these datasets by using the Systematic Target OPportunity assessment by Genetic Association Predictions (STOPGAP) [86] analysis pipeline that enables merging different GWAS datasets and calculating their linkage disequilibrium (LD) to capture a wider spectrum of relevant genetic signals. While STOPGAP offers already processed datasets, we have used the pipeline in our workflow to process the most recent datasets from the above-mentioned sources. All the data processed with STOPGAP were downloaded on 2nd March 2019.

#### Gene expression data

We have used two different sources i.e., (i) LINCS and (ii) Open Targets to collect gene expression datasets for drug perturbations and diseases in our workflow, respectively. The LINCS dataset is a collection of gene expression signatures obtained by exposing cells to a wide variety of known and novel perturbing agents following the L1000 assay. This dataset was retrieved from the Harmonizome database [87] since it provides an already processed version of the original datasets with more convenient attribute tables that define significant associations between genes and attributes such as cell lines, drugs, and dose information. Furthermore, we made use of Open Targets, a platform that brings together multiple data types by comprehensive and robust data integration from many public databases. It has been widely used for investigations on target identification and prioritization. We have retrieved gene expression signatures data for different diseases using the Open Target’s RESTful API on the 5th of March, 2019. Finally, to demonstrate the scalability of PS4DR, we provide the source code to run the workflow with CREEDS [88], an analogous dataset to the two used as case scenarios in the manuscript (https://github.com/ps4dr/ps4dr/tree/master/data/creeds).

### Data preprocessing

**Fig. 3:**
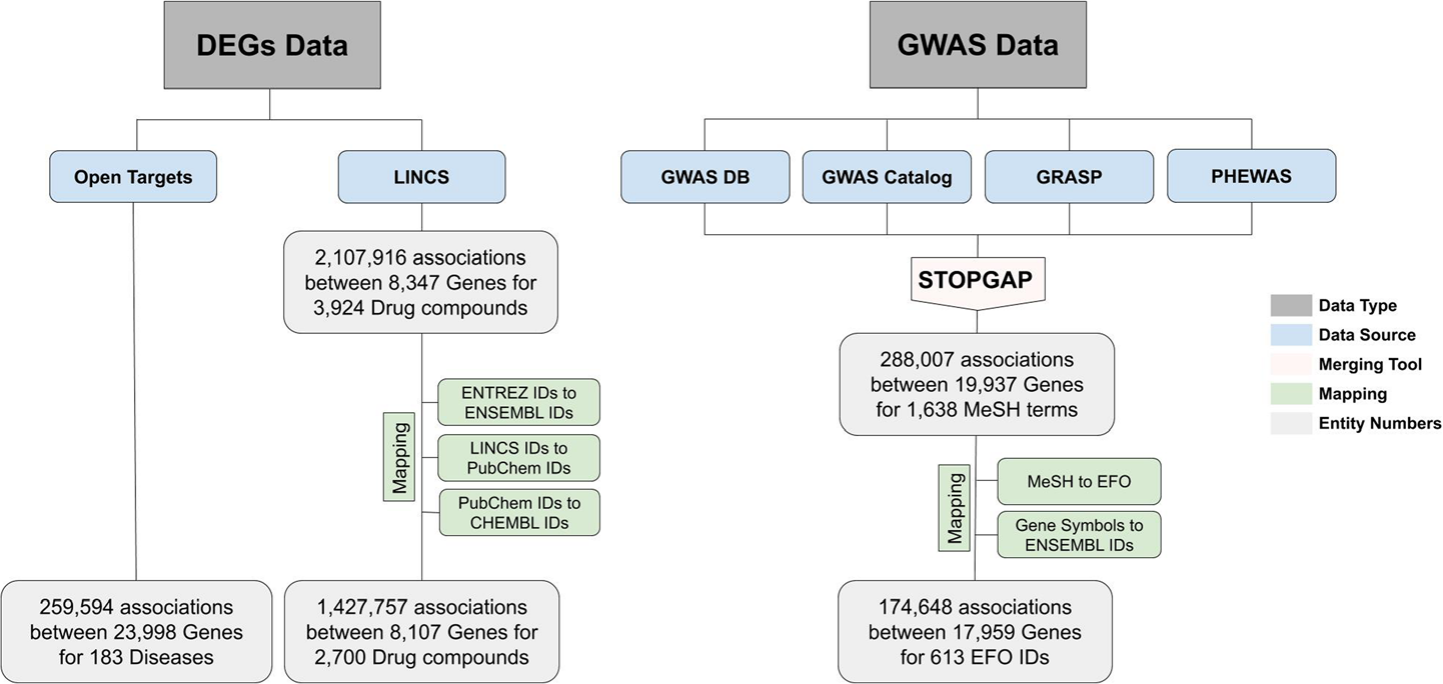
Data preprocessing workflow. This workflow describes the preprocessing of gene expression signatures (left side) and GWAS data (right side) to make them interoperable, as well as the primary and final outcome after the preprocessing. Preprocessing steps include multiple intermediary mappings to get common identifiers for Genes (ENSEMBL identifiers), chemicals (ChEMBL identifiers) and diseases (EFO identifiers)

Since the workflow utilizes a large number of datasets coming from multiple resources in the two data modalities (i.e., genome-wide association data and gene expression signatures) used in the workflow, a series of preprocessing steps were required to harmonize the data to make them interoperable (Fig. 3).

We harmonized Medical Subject Headings (MeSH) [89] concepts used in GWAS studies to facilitate interoperability with the DEG data from Open Targets that exclusively uses the Experimental Factor Ontology (EFO [90]) to catalog disorders. Similarly, we used Ensembl identifiers as the overarching nomenclature that harmonizes all different gene identifiers (e.g., HGNC, Entrez Gene, etc.) in the multiple datasets. The mappings from MeSH to EFO terms were performed using the EFO ontology (version: 2.105). The conversion from different gene identifiers to Ensembl IDs was conducted with the Ensembl release 97 with the biomaRt R package [91]. Finally, LINCS compound identifiers were mapped to PubChem compound identifiers using the mapping table provided by the Ma’ayan Laboratory (http://amp.pharm.mssm.edu/static/hdfs/harmonizome/data/lincscmapchemical/gene_attribute_edges.txt.gz) and then from PubChem compound identifiers to ChEMBL identifiers using UniChem’s RESTful API [92].

These preprocessing steps enabled us to retrieve a total of 174,648 associations between 17,959 genes in 613 diseases from GWAS data. We have used EFO identifiers of these 616 diseases to retrieve their corresponding gene expression signatures in Open Targets using its API. Finally, DEG signatures were fetched for 183 diseases with 259,594 associations between 23,998 genes. Moreover, we also retrieved 17,074 associations between 1060 diseases and 2103 drugs from Open Targets which were at least in clinical trial phase I. Finally, we obtained 1,427,757 associations between 8107 genes and 2700 perturbing agents from the LINCS dataset.

### Filtering via gene set enrichment

The *PS4DR* workflow contains a series of optional filtering steps that enable identifying the genes in the transcriptomics data that have also been reported in GWAS for the same disease. While this step adds the disease context [27] to the gene expression signatures, we leave the possibility for users to omit this step and directly proceed to the pathway enrichment analysis step. Following, we describe each of the filtering steps that are based on calculating the significance of the overlap between the gene sets of the transcriptomics and GWAS data using Fisher’s Exact test.

#### Disease gene expression signatures and GWAS data

This filtering step is based on calculating the significance of the overlap between gene sets from disease gene expression signatures and GWAS data for each disease pair using Fisher’s Exact test. To adjust for multiple testing, *p*-values were corrected with the Benjamini-Hochberg correction [93], and gene sets with a corrected *p*-value above 0.05 were removed. We obtained 26,214 significantly overlapped disease pair gene sets among all the diseases, while 43 of these gene sets originated from the same diseases. These are the ‘disease-specific gene sets’ from 43 diseases, which are both genetically associated and differentially expressed in the same disease. As previously reported by Ferrero and Agarwal [27], we also observed gene sets from GWAS and transcriptomics data of the same disease are more likely to show a significant overlap compared to gene sets from different diseases (Additional file 1: Fig. S3).

#### Drug gene expression signatures and GWAS data

Using the same strategy as the previous step, we filtered drug perturbed gene expression signatures using GWAS data to retain significantly overlapped gene sets. Here, a more stringently adjusted *p*-value threshold of less than or equal to 1e^− 10^ was used to limit the false positive associations since the drug perturbed data do not have any direct disease context. However, we used additional drug-disease associations retrieved from Open Targets to give disease context, to an extent, to the drug perturbed gene expression signatures. Finally, we obtained 22,551 significantly overlapped gene sets which are genetically associated with a particular disease and also differentially expressed by drug perturbations in the same disease context.

#### Disease gene expression signatures, drug gene expression signatures, and GWAS data

The final step involves further filtering of the resulting gene sets of the two previous filtering steps by applying the same strategy. The aim of this final filtering step is to retrieve drug perturbed differentially expressed gene sets in a disease which are also genetically associated with that same disease. In our case scenario, we obtained 14,631 unique drug-disease pairs with significant gene sets (*q*-value > 0.05) from all possible drug-disease pairs (total number of pairs). These two gene sets (i.e., disease-specific and drug-specific gene sets) will be used in the next step for each disease to identify the drugs that revert the signatures observed in the disease condition.

### Pathway enrichment analysis

We next use pathway enrichment analysis in each disease to calculate the sign of pathway dysregulation (i.e., up- or down-regulation) in both of the input datasets (i.e., disease-specific gene sets and drug-specific gene sets) using one or multiple pathway databases of reference. By running pathway enrichment analysis, we obtain two vectors, one for each input dataset, indicating the sign of dysregulation for each pathway (i.e., up- or down-regulated and no change). Here, it is important to note that pathway enrichment acts as a dimensionality reduction technique by narrowing down the genetic space (on the scale of thousands) to the pathway space (on the scale of hundreds) (Additional file 1: Text Section 4). Although numerous pathway enrichment methods can be applied to the workflow (e.g., GSEA, Signaling Pathway Impact Analysis (SPIA) [94]), the method applied must ultimately provide the sign of pathway dysregulation since this information will be used in the following step for drug prioritization.

**Fig. 4:**
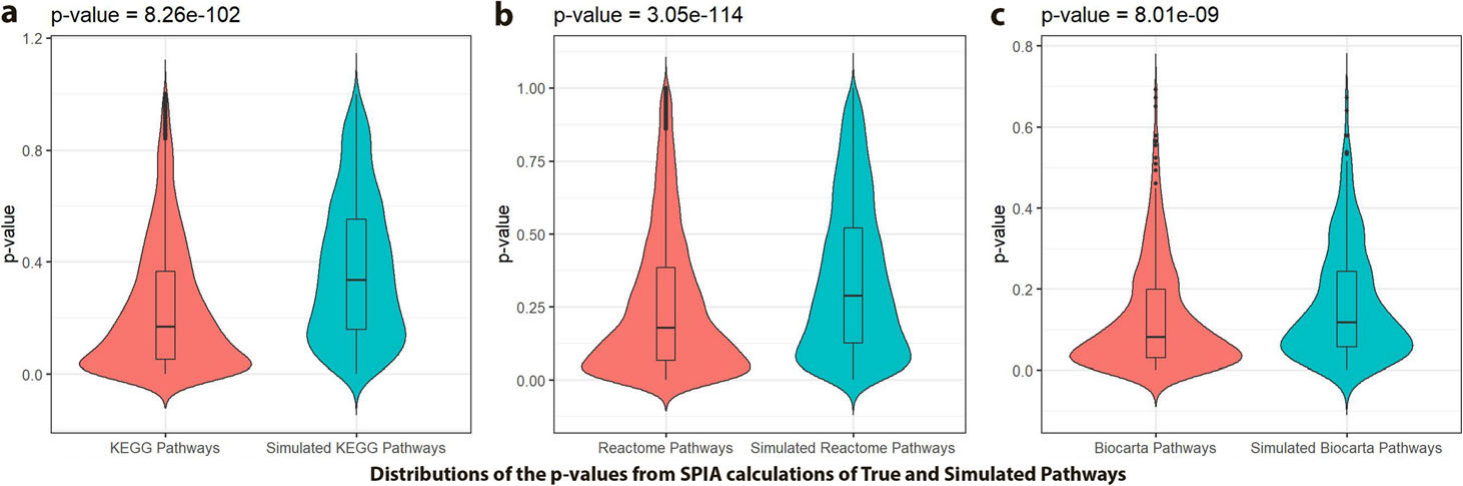
Distributions of the *p*-values resulting from SPIA true and simulated pathways represented as violin plots for a) KEGG, b) Reactome, and c) Biocarta pathway databases. Mann-Whitney U test confirmed that the distributions are significantly different for all three pathway databases (KEGG: *p*-value = 8.26e^-102,^ Reactome: *p*-value = 3.05e^− 114^, Biocarta: *p*-value = 8.01e^− 09^). These results demonstrate that while true pathways yield meaningful results (i.e., lower *p*-values), simulated pathways are rarely significantly enriched

Here, we demonstrate the workflow using one of the most popular topology-based enrichment methods, SPIA, on three pathway databases (i.e., KEGG [95]; Reactome [96]; and Biocarta [97]). Since SPIA requires the pathway input files in a specific binary matrix format, we have used two different tools to prepare pathway datasets for SPIA input. The SPIA package already provides a function to prepare the pathway input file for KEGG’s KGML files. Therefore, we have downloaded the latest KGML files from KEGG’s ftp site on 27 June 2019 and used the SPIA function ‘makeSPIAdata’ to convert them to the SPIA required input format. However, this function only works with the KGML file format, which is a modified XML used by KEGG. Therefore, we used *graphite* (v 1.30.0 - release 2019-04-17) [98] to create additional pathway input files for SPIA calculations. First, we retrieved the Reactome and Biocarta pathway files by using the graphite function ‘pathways’ and then we prepared SPIA input files of these two databases by using another function, ‘prepareSPIA’. Both these data sets were time-stamped with 2019-04-17. However, as previously mentioned, the workflow could be adapted to employ other pathway enrichment analysis methods such as GSEA (Additional file 1: Text Section 2). First, we performed SPIA on 43 ‘disease-specific gene sets’ in order to evaluate signed pathway dysregulation in a disease context. Next, we conducted SPIA for ‘drug-specific gene sets in disease’ which gives signed pathway dysregulation for all available approved drugs and those under clinical trial in each of 43 diseases. Moreover, to evaluate whether SPIA results can be statistically significant, we performed SPIA with the simulated pathways created using the genes from KEGG, Reactome, and Biocarta. The results of SPIA from these randomly simulated pathway constructs rarely yielded significant up- or down-regulated pathways for any of the diseases we tested; thus, this confirms that true pathways are biologically meaningful (Fig. 4).

### Drug prioritization: correlation score

**Fig. 5:**
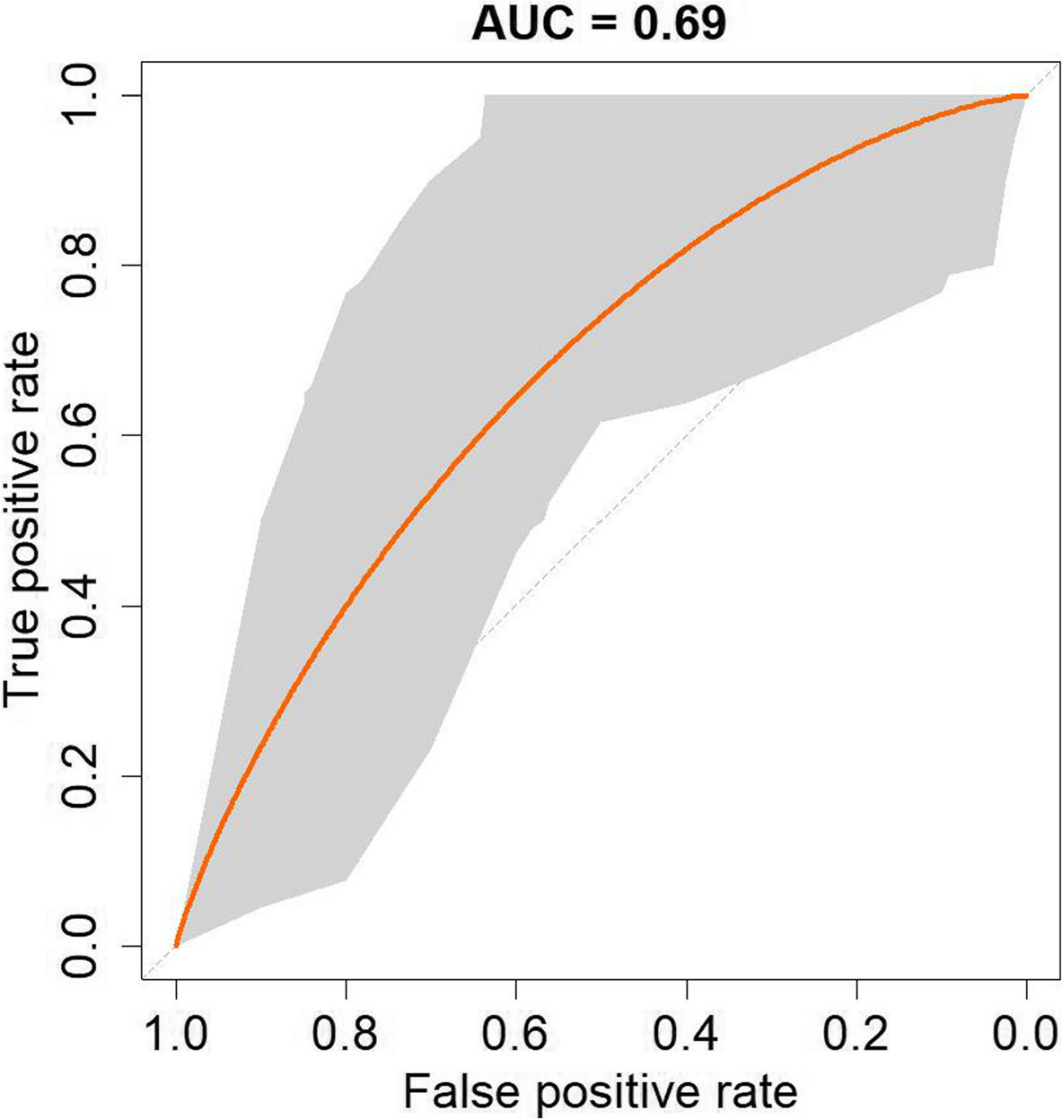
ROC curve of *PS4DR* predicted drugs. ROC curve with 95% confidence interval obtained using existing clinical trials for predicted drugs as positive labels and correlation scores as the ranking metric

The final part of the workflow uses the results of pathway enrichment methods to prioritize drugs based on how well they can counteract the overall pathway signatures on each disease. First, only the statistically significant pathways (*q*-value < 0.05) which are up- or down-regulated in drug and diseases contexts are considered. Next, to facilitate calculating the correlation scores, each affected pathway is assigned with + 1 or − 1 depending on whether it is up- or down-regulated, respectively. Finally, Pearson’s correlation coefficient is calculated using the drug pathway signature vectors against the disease pathway signature vectors. This step results in a list of 26 diseases, while some of the diseases did not have any drugs with a correlation score as the standard deviation was zero for both vectors. Alternatively, Levenshtein distance [99] was also used to calculate the dissimilarity score between the drug and disease pathway signature vectors. We selected arbitrary thresholds for correlation scores (i.e., less than or equal to − 0.4) and affected pathways (i.e., greater than or equal to 50%) to reduce the number of drug candidates in each disease for further manual investigation. However, users can decide the threshold according to their preferences. As a validation step*,* we generated the ROC curve (Fig. 5) for the predicted drug-disease associations by using the correlation scores as predictors and their available clinical trial evidence as labels. The resulting AUC of 0.69 demonstrates that *PS4DR* can prioritize several drugs for given diseases that are already on clinical trials. While we achieved a slightly higher AUC-ROC than Ferrero et al. (AUC-ROC = 0.64), we must note some subtle methodological differences. First, we used a dataset that is 2 years newer than Ferrero et al. (2019 versus 2017). Second, we used anti-correlation scores as the predictor instead of adjusted *p*-values from Fisher’s test for significantly overlapped genesets. Third, we used the same methodology to calculate the AUC, but because of our prioritization, had a smaller number of drug-disease pairs. This was reflected in our wider confidence intervals (0.59–0.82).

### Software and code

R 3.5.1 was used for all data processing and analysis. All code is publicly available at https://github.com/ps4dr/ps4dr under the Apache 2.0 License. Dependencies of the modules used by the workflow and their specific versions are outlined in the repository. Furthermore, we packaged the workflow into a single shell script that can run all the steps with a single command, thus, enabling the reproducibility of the results in the future. Finally, the README file includes an introduction and a tutorial on how to use *PS4DR* and how to add or modify modules within the workflow.

## Supplementary information

**Additional file 1.** This text file contains all supplementary text, tables and figures referenced in the manuscript.

## Data Availability

The datasets generated and/or analyzed during the current study are available in the *PS4DR*’s GitHub repository, [https://github.com/ps4dr/ps4dr]. The datasets generated and/or analyzed during the current study are publicly available at https://github.com/ps4dr/results.

## Abbreviations

AMPK: AMP-activated protein kinase
CKIɛ: Casein Kinase ɛ
CMap: Connectivity Map
CREEDS: CRowd Extracted Expression of Differential Signatures
EFO: Experimental Factor Ontology
FDA: Food and Drug Administration
GSEA: Gene Set Enrichment Analysis
GWAS: Genome-Wide Association Study
LD: Linkage Disequilibrium
LINCS: Library of Integrated Network-Based Cellular Signatures
MeSH: Medical Subject Headings
MoA: Mechanisms of Action
PGC–SCZ: Psychiatric Genomics Consortium–Schizophrenia Workgroup
SLE: Systemic Lupus Erythematosus
SNP: Single nucleotide polymorphism
SPIA: Signaling Pathway Impact Analysis
VEGFR: Vascular Endothelial Growth Factor Receptor
XIAP: X-linked inhibitor of apoptosis protein
