# Supplementary material for: PS4DR: a multimodal workflow for identification and prioritization of drugs based on pathway signatures
Source: BMC Bioinformatics. 2020 Jun 5;21:231. doi: 10.1186/s12859-020-03568-5 (PMC7275349; doi:10.1186/s12859-020-03568-5)
Supplement: Supplementary file 1 — Additional file 1. This text file contains all supplementary text, tables and figures referenced in the manuscript. [file 12859_2020_3568_MOESM1_ESM.docx]

Additional File

# **Supplementary Text Outline**

1. Converter for Generic Networks to SPIA analysis
2. Using other Pathway Enrichment Analysis in the Workflow

# Methodology Comparison

# Additional Value of the Pathway Layer

# **Supplementary Tables Outline**

1. Table S1. Top shortlisted drugs predicted by PS4DR
2. Table S2: Drug repositioning candidates from combined drugs strategy for breast cancer

# **Supplementary Figures Outline**

1. Figure S1: Distribution plots of the correlation scores of the drugs in all the diseases
2. Figure S2: Q-Q plots showing correlation scores for all the investigated diseases
3. Figure S3. Distributions of the p-values of the gene sets from the same and different diseases represented as a boxplot
4. Figure S4. Gene intersections among the disease and pathway databases

# **Supplementary Text**

# **Converter for Generic Networks to SPIA analysis**

In order to facilitate the use of other pathway databases in our workflow, we implemented a converter to SPIA that enables us to conduct this analysis with generic pathway networks. This converter is a new module part of the PathMe package (<https://github.com/PathwayMerger/PathMe>) that can create input files for SPIA by running a simple command: *python -m pathme export spia*. Similar to the graphite R package (<https://github.com/cran/graphite>), PathMe processes generic networks in a way that complex nodes (i.e., reactions and complexes) are flattened, protein and RNAs are collapsed to genes as well as variants (for more information we defer to the PathMe publication). Ultimately, the networks are stored as SPIA input files, namely a connectivity matrix filled with 1s, in the case of a relationship between two nodes, or 0s otherwise. This connectivity matrix spans over multiple relationships (thus, has multiple dimensions) that can directly be mapped from the generic network to the SPIA custom format (see PyBEL-Tools SPIA module for more details; <https://pybel-tools.readthedocs.io/en/latest/algorithms/spia.html>).

# **Using other Pathway Enrichment Analysis in the Workflow**

The following Jupyter Notebook illustrates the use of GSEA in the workflow: <https://github.com/ps4dr/ps4dr/blob/master/notebooks/gsea_alternative.ipynb>. While we used SPIA as our main pathway enrichment analysis tool, any other pathway enrichment analysis could be applied to the workflow as shown in this notebook.

# **Methodology Comparison**

Due to the novelty of our work, we wanted to compare the results generated by our workflow with existing multimodal methodologies for similar tasks. Therefore, we compared our candidate drugs with the ones proposed by the methodology described by Ferrero and Agarwal (2018) applied to the two datasets we have presented. The comparison is presented in the table below and is based on the relative number of drugs recovered by PS4DR that were also hypothesized as candidate drugs by Ferrero and Agarwal’s approach. The results show that the majority of the diseases have a large overlap of candidate drugs. Finally, we would like to point out that while our approach yielded a total of 115 candidate drugs after the filtering step, the compared approach presented a total of 1854 drug-disease associations for the compared diseases. Thus, our approach narrows down the list to a fewer number of candidate drugs and facilitates the prioritization.

| **Disease** | **# Drugs identified by PS4DR** | **# PS4DR drugs present in Ferrero and Agarwal** | **Relative coverage (%)** |
| --- | --- | --- | --- |
| Alzheimer's Disease | 14 | 6 | 42.86 |
| Crohn's Disease | 2 | 1 | 50 |
| Diabetes Mellitus | 5 | 2 | 40 |
| Prostate Adenocarcinoma | 11 | 5 | 45.45 |
| Melanoma | 9 | 5 | 55.56 |
| Celiac Disease | 18 | 6 | 33.33 |
| Type I Diabetes Mellitus | 2 | 1 | 50 |
| Osteoarthritis | 5 | 4 | 80 |
| Parkinson's Disease | 4 | 2 | 50 |
| Systemic Lupus Erythematosus | 9 | 3 | 33.33 |
| Breast Carcinoma | 3 | 2 | 66.67 |
| Multiple Sclerosis | 9 | 4 | 44.44 |

# **Additional Value of the Pathway Layer**

To demonstrate the benefit of a pathway-centric approach with respect to one that directly utilizes differentially expressed signatures, we compared the number of drug hits after calculating the anticorrelation scores using pathways versus using differently expressed signatures on the same multimodal data, respectively. The number of drug hits using differentially expressed signatures was significantly less than the number of drugs hit using the standard pathway approach after applying the same thresholds (i.e., anticorrelation score < -0.4) with only 41 drugs for six indications. Furthermore, we replicated the experiment after filtering out genes with differential expression values smaller than 2 (i.e., |logFC| < 2), a standard filter used for contrast experiments. This additional filtering step is intended to restrict the downstream analysis to genes that are highly dysregulated by filtering genes showing minimal dysregulation which could influence the final anticorrelation score. After applying this step, the number of hits dramatically reduced to two drugs for one single indication. The source code for this analysis is located at <https://github.com/ps4dr/ps4dr/blob/master/R/analysis/geneVsPathwayCentricComparison.R>.

| **Disease** | **Drug** | **Correlation Score** | **Dissimilarity Score** | **Affected Pathway(%)** |
| --- | --- | --- | --- | --- |
| Alzheimer's Disease | Sirolimus | -0.69 | 86.36 | 66.67 |
| Alzheimer's Disease | Pevonedistat | -0.66 | 85 | 60.61 |
| Alzheimer's Disease | Nilotinib | -0.64 | 85 | 60.61 |
| Alzheimer's Disease | Terfenadine | -0.57 | 84.21 | 57.58 |
| Alzheimer's Disease | Doxylaminesuccinate | -0.57 | 83.33 | 54.55 |
| Alzheimer's Disease | Halcinonide | -0.57 | 82.35 | 51.52 |
| Alzheimer's Disease | Promazinehydrochloride | -0.53 | 77.27 | 66.67 |
| Alzheimer's Disease | Mosapride | -0.45 | 75 | 60.61 |
| Alzheimer's Disease | Pimozide | -0.45 | 73.68 | 57.58 |
| Alzheimer's Disease | Ritanserin | -0.45 | 73.68 | 57.58 |
| Alzheimer's Disease | Betamethasone | -0.44 | 77.27 | 66.67 |
| Alzheimer's Disease | Cinacalcethydrochloride | -0.43 | 75 | 72.73 |
| Alzheimer's Disease | Methapyrilenehydrochloride | -0.43 | 75 | 72.73 |
| Alzheimer's Disease | Trametinib | -0.4 | 80 | 60.61 |
| Breast Carcinoma | At-7519 | -0.48 | 77.03 | 86.05 |
| Breast Carcinoma | Omacetaxinemepesuccinate | -0.48 | 73.97 | 84.88 |
| Breast Carcinoma | Rigosertib | -0.44 | 73.85 | 75.58 |
| Celiac Disease | Acitretin | -0.46 | 69.23 | 59.09 |
| Celiac Disease | Albendazole | -0.46 | 69.23 | 59.09 |
| Celiac Disease | Bms-777607 | -0.46 | 69.23 | 59.09 |
| Celiac Disease | Celecoxib | -0.46 | 69.23 | 59.09 |
| Celiac Disease | Dequalinium | -0.46 | 69.23 | 59.09 |
| Celiac Disease | Donepezilhydrochloride | -0.46 | 69.23 | 59.09 |
| Celiac Disease | Lexibulin | -0.46 | 69.23 | 59.09 |
| Celiac Disease | Linifanib | -0.46 | 69.23 | 59.09 |
| Celiac Disease | Lovastatin | -0.46 | 69.23 | 59.09 |
| Celiac Disease | Maprotiline | -0.46 | 69.23 | 59.09 |
| Celiac Disease | Mebendazole | -0.46 | 69.23 | 59.09 |
| Celiac Disease | Panobinostat | -0.46 | 69.23 | 59.09 |
| Celiac Disease | Pazopanibhydrochloride | -0.46 | 69.23 | 59.09 |
| Celiac Disease | Podofilox | -0.46 | 69.23 | 59.09 |
| Celiac Disease | Tenofovir | -0.46 | 69.23 | 59.09 |
| Celiac Disease | Gsk-461364 | -0.43 | 68.75 | 72.73 |
| Celiac Disease | Canertinib | -0.41 | 64.29 | 63.64 |
| Celiac Disease | Fluticasonepropionate | -0.41 | 64.29 | 63.64 |
| Chronic Obstructive Pulmonary Disease | Doxorubicinhydrochloride | -0.5 | 70 | 71.43 |
| Crohn's Disease | Pimasertib | -0.45 | 90.48 | 52.5 |
| Crohn's Disease | Topotecanhydrochloride | -0.42 | 82.61 | 57.5 |
| Diabetes Mellitus | Palbociclib | -0.58 | 75 | 50 |
| Diabetes Mellitus | Ruxolitinib | -0.58 | 75 | 50 |
| Diabetes Mellitus | Atomoxetinehydrochloride | -0.41 | 60 | 62.5 |
| Diabetes Mellitus | Nonoxynol-9 | -0.41 | 60 | 62.5 |
| Diabetes Mellitus | Promazinehydrochloride | -0.41 | 60 | 62.5 |
| Glioblastoma Multiforme | Aminosalicylicacid | -0.84 | 95.24 | 72.41 |
| Glioblastoma Multiforme | Etazolate | -0.84 | 95.24 | 72.41 |
| Glioblastoma Multiforme | Hydroxychloroquine | -0.46 | 85 | 68.97 |
| Glioblastoma Multiforme | Ropinirole | -0.46 | 85 | 68.97 |
| Lung Carcinoma | Fedratinib | -0.53 | 70 | 66.67 |
| Lung Carcinoma | Bi-2536 | -0.41 | 60 | 66.67 |
| Lung Carcinoma | Dasatinib | -0.41 | 60 | 66.67 |
| Melanoma | Crizotinib | -0.64 | 80 | 74.07 |
| Melanoma | Olmesartan | -0.58 | 73.33 | 55.56 |
| Melanoma | Sepantronium | -0.52 | 70 | 74.07 |
| Melanoma | Bortezomib | -0.52 | 70.59 | 62.96 |
| Melanoma | Fluspirilene | -0.5 | 66.67 | 55.56 |
| Melanoma | Azd-2014 | -0.44 | 61.11 | 66.67 |
| Melanoma | Olaparib | -0.44 | 61.11 | 66.67 |
| Melanoma | Tivozanib | -0.44 | 61.11 | 66.67 |
| Melanoma | Belinostat | -0.43 | 60 | 55.56 |
| **Multiple Sclerosis** | **Methylprednisolone** | **-0.57** | **78.26** | **82.14** |
| Multiple Sclerosis | Diflorasonediacetate | -0.56 | 76.19 | 75 |
| Multiple Sclerosis | Bendroflumethiazide | -0.5 | 72.22 | 64.29 |
| Multiple Sclerosis | Pitavastatincalcium | -0.49 | 76.47 | 60.71 |
| Multiple Sclerosis | Lestaurtinib | -0.49 | 75 | 57.14 |
| Multiple Sclerosis | Desoximetasone | -0.48 | 73.33 | 53.57 |
| Multiple Sclerosis | Alvespimycin | -0.47 | 73.33 | 53.57 |
| Multiple Sclerosis | Fluconazole | -0.44 | 80 | 53.57 |
| Multiple Sclerosis | Clocortolonepivalate | -0.43 | 73.33 | 53.57 |
| Osteoarthritis | Canertinib | -1 | 100 | 60 |
| Osteoarthritis | Cytarabine | -1 | 100 | 60 |
| Osteoarthritis | Fedratinib | -1 | 100 | 60 |
| Osteoarthritis | Neratinib | -1 | 100 | 60 |
| Osteoarthritis | Pimasertib | -1 | 100 | 60 |
| Pancreatic Carcinoma | Fenofibrate | -0.58 | 75 | 57.14 |
| Pancreatic Carcinoma | Fluoxetine | -0.58 | 75 | 57.14 |
| Pancreatic Carcinoma | Menadione | -0.58 | 75 | 57.14 |
| Pancreatic Carcinoma | Methyldopa | -0.58 | 75 | 57.14 |
| Pancreatic Carcinoma | Praziquantel | -0.58 | 75 | 57.14 |
| Pancreatic Carcinoma | Tosedostat | -0.58 | 75 | 57.14 |
| Pancreatic Carcinoma | Venlafaxine | -0.58 | 75 | 57.14 |
| Pancreatic Carcinoma | Azd-6482 | -0.41 | 60 | 71.43 |
| Parkinson's Disease | Amlodipine | -0.47 | 71.43 | 53.85 |
| Parkinson's Disease | Decitabine | -0.47 | 71.43 | 53.85 |
| Parkinson's Disease | Paroxetinehydrochloride | -0.47 | 71.43 | 53.85 |
| Parkinson's Disease | Sulconazolenitrate | -0.47 | 71.43 | 53.85 |
| Prostate Adenocarcinoma | Alprostadil | -1 | 100 | 50 |
| Prostate Adenocarcinoma | Alvocidib | -1 | 100 | 50 |
| Prostate Adenocarcinoma | Azd-5438 | -1 | 100 | 50 |
| Prostate Adenocarcinoma | Azd-8055 | -1 | 100 | 50 |
| Prostate Adenocarcinoma | Dasatinib | -1 | 100 | 50 |
| Prostate Adenocarcinoma | Gamolenicacid | -1 | 100 | 50 |
| Prostate Adenocarcinoma | Halcinonide | -1 | 100 | 50 |
| Prostate Adenocarcinoma | Linifanib | -1 | 100 | 50 |
| Prostate Adenocarcinoma | Mibefradil | -1 | 100 | 50 |
| Prostate Adenocarcinoma | Niclosamide | -1 | 100 | 50 |
| Prostate Adenocarcinoma | Prednicarbate | -1 | 100 | 50 |
| Rheumatoid Arthritis | Enclomiphenecitrate | -0.5 | 70 | 58.82 |
| Squamous Cell Carcinoma | Gsk-1070916 | -1 | 100 | 66.67 |
| Squamous Cell Carcinoma | Tozasertib | -1 | 100 | 66.67 |
| Systemic Lupus Erythematosus | Rigosertib | -0.86 | 94.44 | 94.74 |
| Systemic Lupus Erythematosus | Tivozanib | -0.77 | 90.91 | 57.89 |
| Systemic Lupus Erythematosus | Diflorasonediacetate | -0.76 | 88.89 | 94.74 |
| Systemic Lupus Erythematosus | Atomoxetinehydrochloride | -0.68 | 89.47 | 100 |
| Systemic Lupus Erythematosus | Triclosan | -0.68 | 88.89 | 94.74 |
| Systemic Lupus Erythematosus | Mibefradil | -0.58 | 86.67 | 78.95 |
| Systemic Lupus Erythematosus | Metergoline | -0.56 | 83.33 | 94.74 |
| Systemic Lupus Erythematosus | Oxfendazole | -0.56 | 83.33 | 94.74 |
| Systemic Lupus Erythematosus | Epirubicinhydrochloride | -0.52 | 81.82 | 57.89 |
| Type I Diabetes Mellitus | Dasatinib | -0.45 | 62.5 | 66.67 |
| Type I Diabetes Mellitus | Tinidazole | -0.45 | 66.67 | 50 |
| Type II Diabetes Mellitus | Ci-1040 | -1 | 100 | 75 |
| Type II Diabetes Mellitus | Brinzolamide | -0.5 | 66.67 | 75 |
| Type II Diabetes Mellitus | Canertinib | -0.5 | 66.67 | 75 |
| Type II Diabetes Mellitus | Gsk-461364 | -0.5 | 66.67 | 75 |
| Type II Diabetes Mellitus | Lidoflazine | -0.5 | 66.67 | 75 |

**Table S1. Top shortlisted drugs predicted by PS4DR.** All shortlisted drugs for 19 diseases which are predicted by our workflow that met our arbitrary correlation coefficient score thresholds of less than equal to - 0.4 and affected pathways (%) more than equal to 50.

| **Drug_1** | **Drug_2** | **Correlation Score** | **Dissimilarity Score** | **Affected Pathway(%)** |
| --- | --- | --- | --- | --- |
| AT-7519 | Axitinib | -0.6 | 81.08 | 86.05 |
| AT-7519 | Azd-1775 | -0.51 | 74.03 | 89.53 |
| AT-7519 | BGJ-398 | -0.54 | 77.92 | 89.53 |
| AT-7519 | BMS-777607 | -0.53 | 75.68 | 86.05 |
| AT-7519 | Brimonidine | -0.58 | 78.95 | 88.37 |
| AT-7519 | Bromhexine | -0.55 | 77.63 | 88.37 |
| AT-7519 | Hexylcaine | -0.53 | 77.33 | 87.21 |
| AT-7519 | Iopanoic Acid | -0.52 | 76.32 | 88.37 |
| AT-7519 | Isotretinoin | -0.53 | 77.03 | 86.05 |
| AT-7519 | Ketorolac | -0.54 | 74.32 | 86.05 |
| AT-7519 | Levocetirizine | -0.54 | 77.33 | 87.21 |
| AT-7519 | Megestrol Acetate | -0.53 | 77.03 | 86.05 |
| AT-7519 | Nadolol | -0.59 | 79.73 | 86.05 |
| AT-7519 | PF-04217903 | -0.52 | 77.33 | 87.21 |
| AT-7519 | R-406 | -0.51 | 70 | 93.02 |
| AT-7519 | Rigosertib | -0.51 | 72.37 | 88.37 |
| AT-7519 | Selumetinib | -0.57 | 79.49 | 90.7 |
| AT-7519 | TAK-715 | -0.58 | 79.73 | 86.05 |
| Omacetaxine Mepesuccinate | Axitinib | -0.59 | 78.08 | 84.88 |
| Omacetaxine Mepesuccinate | AZD-1775 | -0.51 | 71.79 | 90.7 |
| Omacetaxine Mepesuccinate | BMS-777607 | -0.52 | 72.97 | 86.05 |
| Omacetaxine Mepesuccinate | Brimonidine | -0.58 | 75.68 | 86.05 |
| Omacetaxine Mepesuccinate | Bromhexine | -0.52 | 74.32 | 86.05 |
| Omacetaxine Mepesuccinate | Erlotinib Hydrochloride | -0.51 | 71.43 | 89.53 |
| Omacetaxine Mepesuccinate | Hexylcaine | -0.53 | 74.67 | 87.21 |
| Omacetaxine Mepesuccinate | Iopanoic Acid | -0.54 | 74.67 | 87.21 |
| Omacetaxine Mepesuccinate | Isotretinoin | -0.55 | 75.34 | 84.88 |
| Omacetaxine Mepesuccinate | Ketorolac | -0.51 | 69.86 | 84.88 |
| Omacetaxine Mepesuccinate | Levocetirizine | -0.54 | 74.32 | 86.05 |
| Omacetaxine Mepesuccinate | Nadolol | -0.61 | 78.08 | 84.88 |
| Omacetaxine Mepesuccinate | PF-04217903 | -0.51 | 73.33 | 87.21 |
| Omacetaxine Mepesuccinate | Rigosertib | -0.55 | 73.68 | 88.37 |
| Omacetaxine Mepesuccinate | TAK-715 | -0.57 | 76.71 | 84.88 |
| Omacetaxine Mepesuccinate | Tivantinib | -0.51 | 67.57 | 86.05 |

**Table S2: Drug repositioning candidates from combined drugs strategy for breast cancer.** This table lists all drug pairs from the combined drugs strategy in breast cancer that met our arbitrary correlation coefficient score thresholds of less than equal to - 0.5 and affected pathways (%) more than equal to 80%.

**
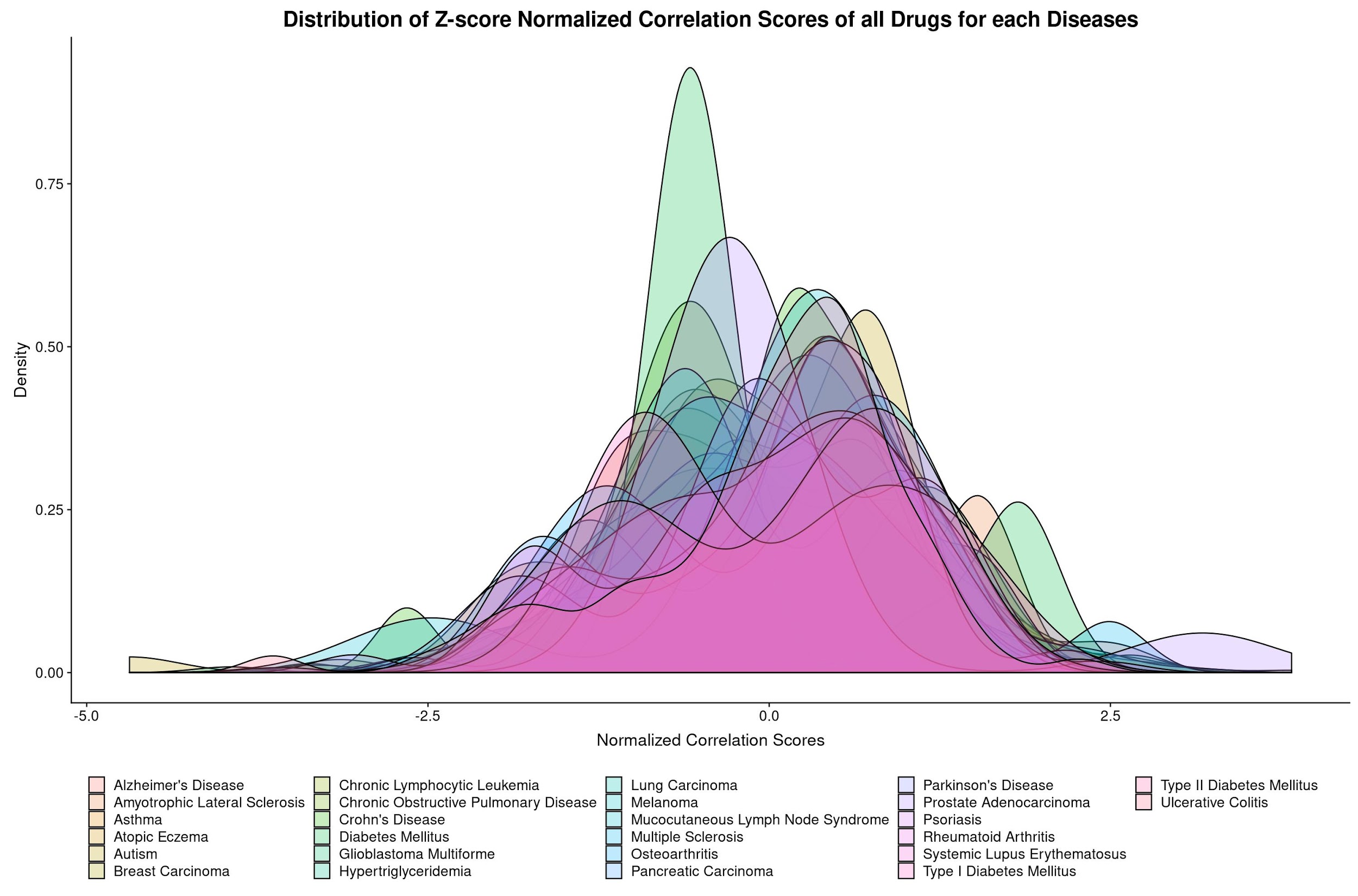
**

**Figure S1: Distribution plots of the correlation scores of the drugs in all the diseases.** Distributions of Z-score normalized correlation scores for all drugs in each disease. Correlation scores for 26 diseases are plotted along the x-axis and density distribution on the y-axis. Drugs with higher negative correlation scores in each disease are of potential interest for repositioning. On the other hand, drugs with positive correlation could be used as a candidate for creating disease cell lines.

**
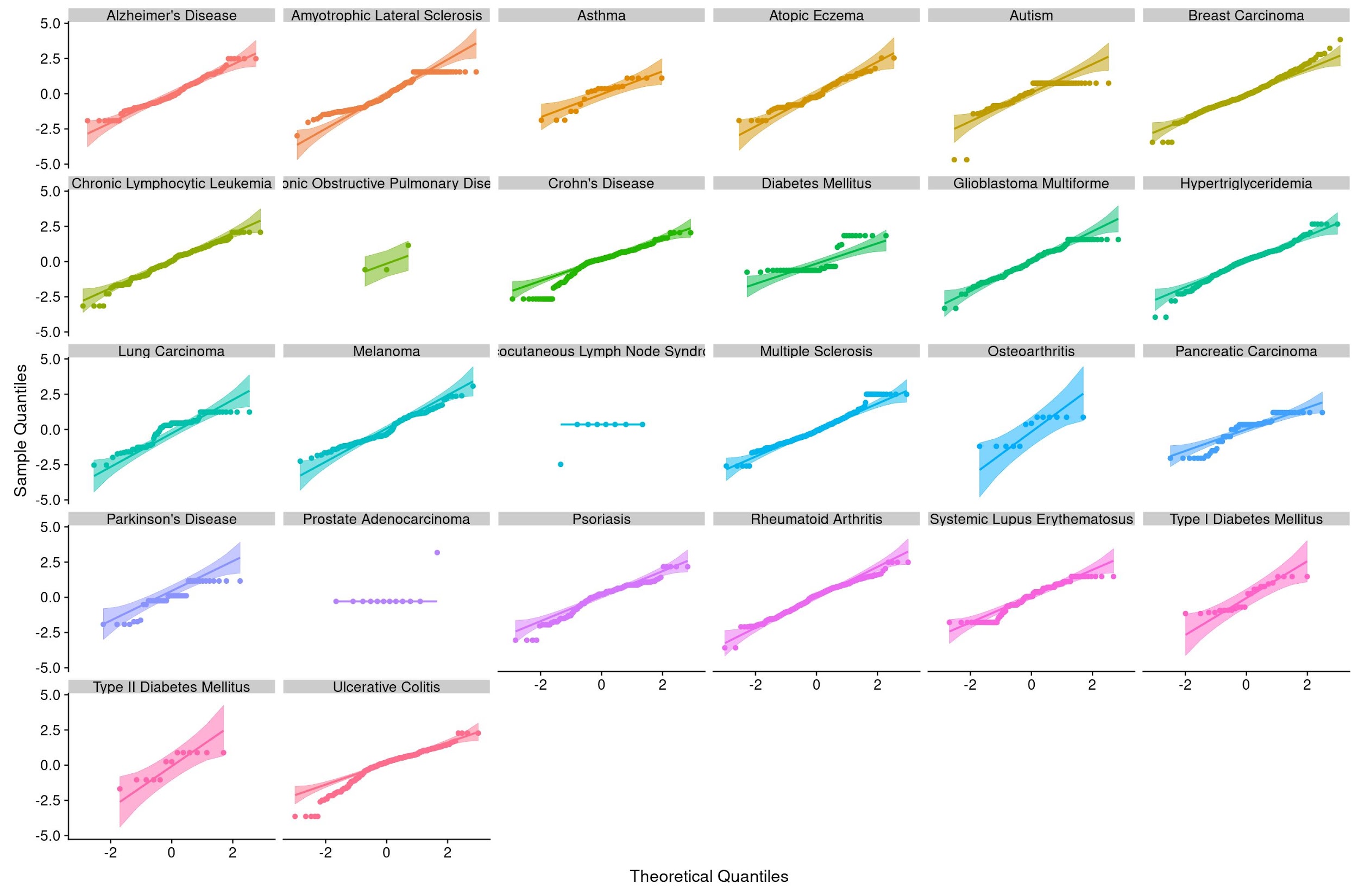
**

**Figure S2: Q-Q plots showing correlation scores for all the investigated diseases.** Although some diseases such as prostate adenocarcinoma or osteoarthritis do not show a normal distribution, the majority of the diseases show normally distributed correlation scores.


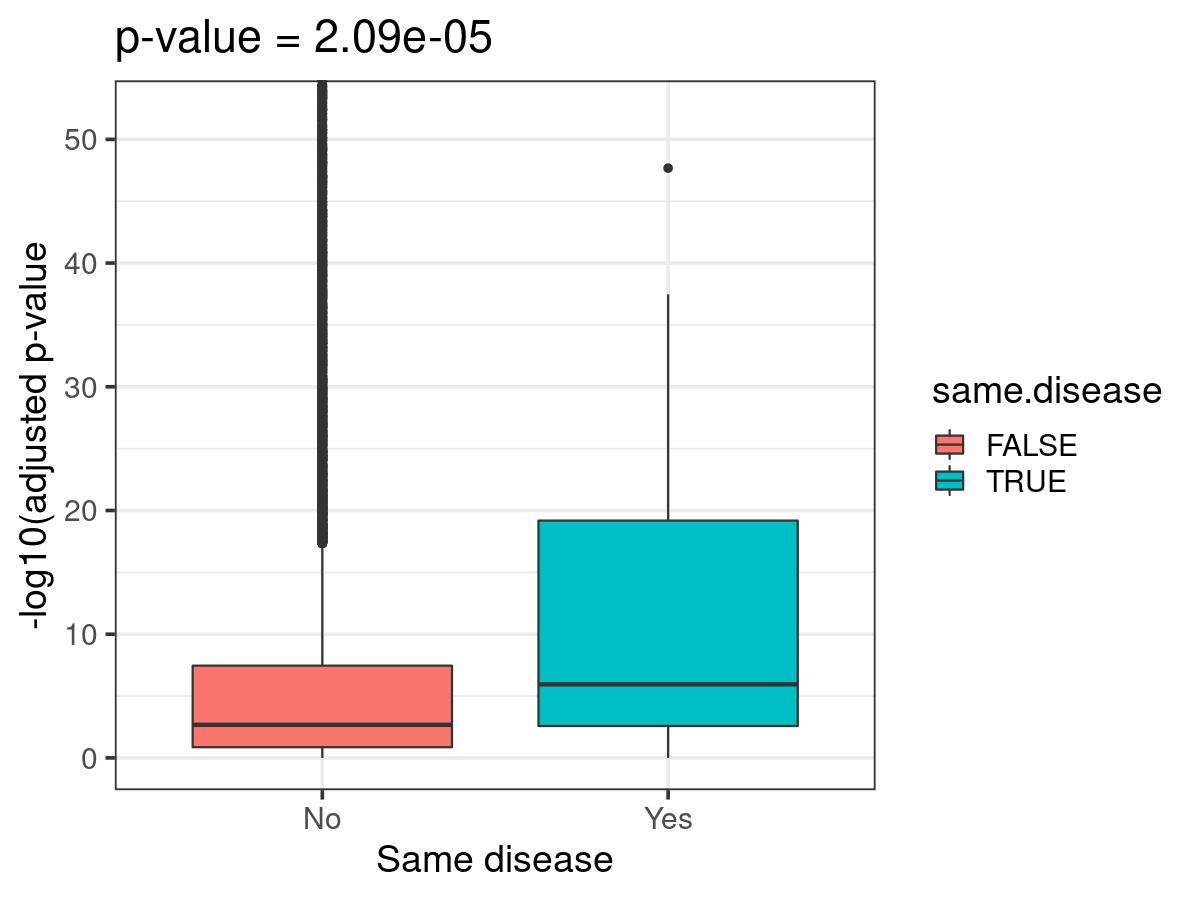


**Figure S3. Distributions of the *p*-values of the gene sets from the same and different diseases represented as a boxplot.** Mann-Whitney U test confirmed that the distributions are significantly different (*p*-value=2.09e^-5^). These results demonstrate that genes which are genetically associated with the disease (i.e., via GWAS) are more likely to be enriched for genes differentially expressed plots in the same disease, in concordance with Ferrero and Agarwal (2018).

**
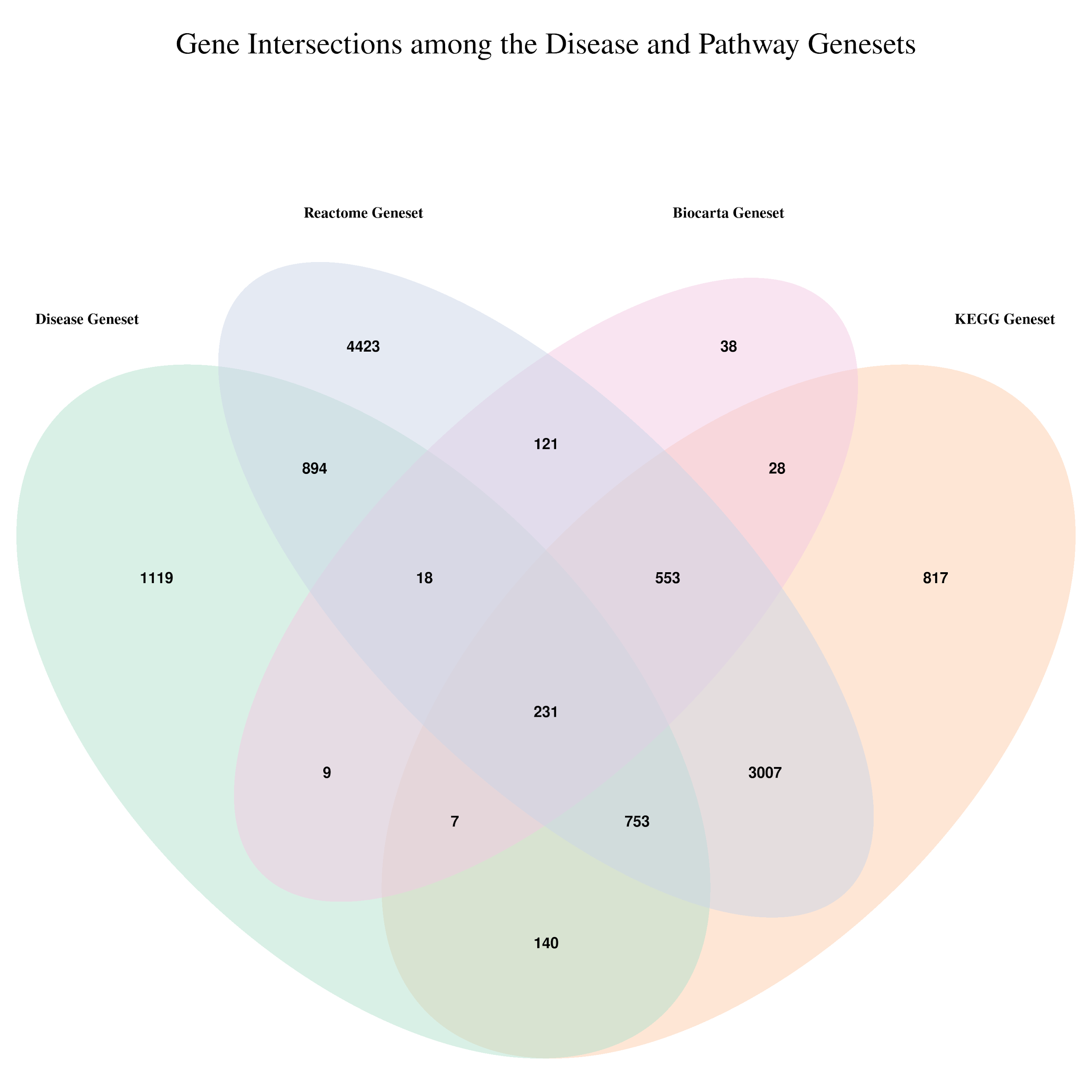
**

**Figure S4. Gene intersections among the disease and pathway databases.** This Venn diagram depicts the overlap between the genes from the final 43 diseases that have both DEGs and GWASs data in our workflow and the genes in each of the three pathway databases (i.e., KEGG, Reactome, and Biocarta).
